# Immune infiltrate diversity confers a good prognosis in follicular lymphoma

**DOI:** 10.1007/s00262-021-02945-0

**Published:** 2021-04-30

**Authors:** Anna-Maria Tsakiroglou, Susan Astley, Manàs Dave, Martin Fergie, Elaine Harkness, Adeline Rosenberg, Matthew Sperrin, Catharine West, Richard Byers, Kim Linton

**Affiliations:** 1 Division of Cancer Sciences, Manchester Academic Health Science Centre, University of Manchester, Manchester, UK; 2 Manchester Cancer Research Centre, Wilmslow Road, Manchester, M20 4QL UK; 3 Division of Informatics, Imaging and Data Sciences, School of Health Sciences, University of Manchester, Manchester, UK; 4 Prevent Breast Cancer and Nightingale Breast Screening Centre, Wythenshawe Hospital, Manchester University NHS Foundation Trust, Manchester, UK; 5 Division of Dentistry, Manchester Academic Health Science Centre School of Medical Sciences, University of Manchester, Manchester, UK; 6 School of Medical Sciences, University of Manchester, Manchester, UK; 7 The Christie NHS Foundation Trust, Manchester, UK; 8 Manchester Royal Infirmary, Manchester University NHS Foundation Trust (MFT), Oxford Road, Manchester, M13 9WL UK

**Keywords:** Tumour microenvironment, Multiplex, Follicular lymphoma, Diversity, Spatial heterogeneity, Prognosis

## Abstract

**Background:**

Follicular lymphoma (FL) prognosis is influenced by the composition of the tumour microenvironment. We tested an automated approach to quantitatively assess the phenotypic and spatial immune infiltrate diversity as a prognostic biomarker for FL patients.

**Methods:**

Diagnostic biopsies were collected from 127 FL patients initially treated with rituximab-based therapy (52%), radiotherapy (28%), or active surveillance (20%). Tissue microarrays were constructed and stained using multiplex immunofluorescence (CD4, CD8, FOXP3, CD21, PD-1, CD68, and DAPI). Subsequently, sections underwent automated cell scoring and analysis of spatial interactions, defined as cells co-occurring within 30 μm. Shannon’s entropy, a metric describing species biodiversity in ecological habitats, was applied to quantify immune infiltrate diversity of cell types and spatial interactions. Immune infiltrate diversity indices were tested in multivariable Cox regression and Kaplan–Meier analysis for overall (OS) and progression-free survival (PFS).

**Results:**

Increased diversity of cell types (HR = 0.19 95% CI 0.06–0.65, *p* = 0.008) and cell spatial interactions (HR = 0.39, 95% CI 0.20–0.75, *p* = 0.005) was associated with favourable OS, independent of the Follicular Lymphoma International Prognostic Index. In the rituximab-treated subset, the favourable trend between diversity and PFS did not reach statistical significance.

**Conclusion:**

Multiplex immunofluorescence and Shannon’s entropy can objectively quantify immune infiltrate diversity and generate prognostic information in FL. This automated approach warrants validation in additional FL cohorts, and its applicability as a pre-treatment biomarker to identify high-risk patients should be further explored. The multiplex image dataset generated by this study is shared publicly to encourage further research on the FL microenvironment.

**Supplementary Information:**

The online version contains supplementary material available at 10.1007/s00262-021-02945-0.

## Introduction

In follicular lymphoma (FL) there is a clinical need for pre-treatment identification of high-risk patients, recognisable as the subset of patients (15–30%) with early progression and poor survival outcomes despite therapeutic advances [1–3]. Current FL prognostic biomarkers, such as the Follicular Lymphoma International Prognostic Index (FLIPI) [4, 5], are well validated but lack the necessary precision for clinical decision-making. To improve risk stratification, subsets of tumour-infiltrating lymphocytes (TILs) have been studied, but there is no consensus on the observed effect [1, 6–8]. Immune subsets of prognostic interest include, among others: CD68^+^ lymphoma-associated macrophages [9–12], CD3^+^ T cells [10, 12–14], CD4^+^ T helper cells [12–14], CD8^+^ cytotoxic T cells [12–15], CD4^+^FOXP3^+^ T regulatory cells (Tregs) [12, 14, 16–18], CD21^+^ dendritic cells [12], mast cells [19], and PD-1 expressing T-cells [12, 14, 20]. These cells engage in crosstalk through multiple pathways [21], and therefore a holistic observation of immune infiltrate diversity could be more informative than examining isolated components.

In ecological sciences the Shannon diversity (or entropy) index quantifies biodiversity in terms of “evenness” [22]. For example, if three species are found in an area, and one accounts for 99% of the population, this community would be considered less diverse than one where the three species are found in approximately equal abundances. Entropy is calculated from the proportion of each species in the community and increases when diversity is higher. It has found applications in histopathology to quantify heterogeneity of HER2 expression [23] and chromosome 8q24 copy number variation [24] in breast cancer. If we consider each cell phenotype as a species, this metric can be applied to quantify immune infiltrate diversity.

Similarly, it is possible to quantify the diversity of not only phenotypes but also their spatial interactions, which is recognised for its potential as a biomarker [25] for many tumour types including FL [16, 26]. The hypothesised interactions distribution (HID) method [27] can identify spatial interactions defined as co-occurrences of different cell types within 30 μm. The diversity of these spatial interactions can also be investigated using entropy.

We aimed to develop a methodology to quantitatively assess immune infiltrate diversity in the tumour microenvironment of FL and test its potential utility as a prognostic biomarker. To this end, an automated multiplex immunofluorescence and image analysis pipeline were developed to simultaneously identify cells positive for CD4, CD68, CD8, CD21, FOXP3, and PD-1. We show that increased diversity of immune infiltrate populations and interactions is associated with improved overall survival (OS) in a cohort of FL patients.

## Methods

### Dataset

#### Cohort selection

The study was conducted with approval from the North–West Multi-centre Ethics Committee (03/08/016), according to the Declaration of Helsinki. Examination of 350 FL patients’ electronic records in a random order from the archives of The Christie NHS Foundation Trust, Manchester, UK, identified 262 patients meeting the inclusion criteria: adult; diagnosed from incisional or excisional biopsy; non-primary cutaneous; and treated at first presentation with radiotherapy, watchful waiting, or rituximab-based systemic therapy. Pre-treatment diagnostic biopsies were requested for 262 patients, of which 131 had sufficient tissue. The 131 patients were diagnosed between 1998 and 2015, with a median follow-up of 114 months (range 3–199 months). Histological diagnosis of FL was re-confirmed by an expert haemato-pathologist (R.B). Regions of interest were identified by the haemato-pathologist, and cores were extracted in triplicate from formalin-fixed, paraffin-embedded (FFPE) blocks to construct five tissue microarrays (TMA). Core diameter was 1.2 mm. Follicular and extrafollicular regions were both selected for inclusion in the TMA. A section of each TMA stained with H&E is provided [28], to demonstrate the morphology of selected regions. Some cores were excluded because of poor quality, leaving 349 cores from 130 patients. The OS distribution was similar between the 130-patient cohort and the patients with insufficient tumour material (Supplementary Fig. 1, log rank test *p* = 0.37). Furthermore, three patients with FL grade 3b were excluded from survival analyses, as their disease progression and treatment pathways resemble more closely Diffuse Large B-cell Lymphoma and grade 3b FL is generally considered a separate disease entity [29]. Finally, 342 cores from 127 patients remained available for further analyses.

#### Clinical endpoints

OS was recorded for all patients (*N* = 127). Progression-free survival (PFS) and disease progression within 24 months of starting treatment (POD24) were recorded only for patients treated with rituximab-containing immuno-chemotherapy at first presentation (*N* = 67). The events of disease progression and relapse were defined using the Lugano criteria [30]. PFS was calculated from diagnosis until the first observed progression event (or disease-specific death) or, if no events were observed, until the date of last follow-up.

### Multiplex immunofluorescence imaging

In this section we describe preparation of the image dataset using multiplex immunofluorescence and multi-spectral scanning.

#### Antibody panel selection

A panel of antibodies was selected to identify non-neoplastic immune infiltrate subsets. CD68 was selected to observe monocytic cells and particularly macrophages [10, 11]. CD8 was used to observe cytotoxic T cells (CD8^+^) [8]. T helper cells were identified by inclusion of the CD4 marker, as the subset that expressed CD4, but not CD68 [8]. Additionally, FOXP3 was used to identified T regulatory cells (Tregs [CD4^+^FOXP3^+^]) [26]. The CD21 marker was used to identify follicular dendritic cell areas [31]. While CD21 might also be expressed in B-cells, follicular dendritic cells in follicular lymphoma can be identified by their characteristic meshwork staining pattern [32]. PD-1 detected CD4^+^PD-1^+^ T follicular helper cells and CD8^+^PD-1^+^ lymphocytes [20]. Finally, DAPI (4′,6-diamidino-2-phenylindole) was the nuclear counterstain. No B-cell tumour marker was used, as we aimed to study the diversity of the non-neoplastic microenvironment.

#### Staining protocol

A single 4-μm section was cut from each of the five TMAs of the cohort. These sections were stained with a 6-plex immunofluorescent assay using tyramide signal amplification, the Opal 7 colour kit (Akoya Biosciences, CA, USA), and the Ultra Discovery auto-stainer (Roche, Switzerland), as described in the experimental protocol of Tsakiroglou et al. [33], and are summarised in Supplementary Table 1. The OPAL detection system allows for repeated cycles of staining and stripping of anti-mouse and anti-rabbit antibodies. In each cycle, the primary antibody and secondary conjugated to horseradish peroxidase (HRP) are incubated on the tissue, followed by fluorophore-labelled tyramide. The catalytic reaction that occurs binds the fluorophore strongly on the tissue epitopes. Thereafter, heat stripping is applied to remove the antibody before applying the next one, but the fluorophores remain on the tissue. We quantitatively validated good agreement between single-plex and multiplex immunofluorescent assays (Supplementary Material Sect. 2, Supplementary Fig. 2–5).

#### Image acquisition

**Fig. 1:**
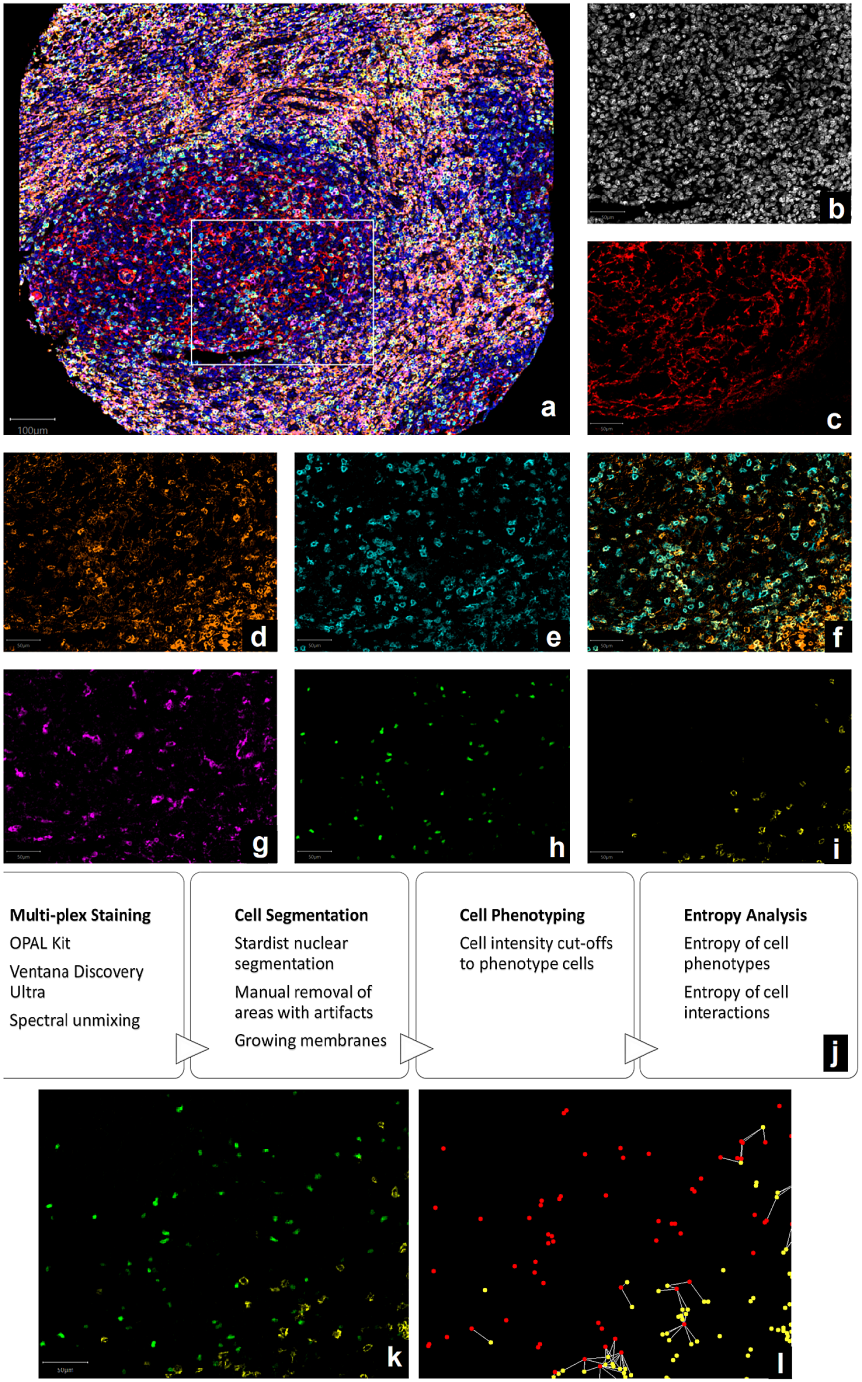
Multiplex immunofluorescence and automated image analysis to measure immune infiltrate diversity. **a** Is a composite multiplex image displaying all stains together using pseudo-colours: DAPI is blue, CD21 is red, CD4 is orange, PD-1 is cyan, CD8 is yellow, CD68 is magenta, and FOXP3 is green. Panels (b-i) and (k-l) show the exact same tissue region, indicated as a white rectangle in (a). Panels (b-i) demonstrate the process of spectral unmixing: **b** shows DAPI in white; **c** shows CD21 in red; **d** shows CD4 in orange; **e** shows PD-1 in cyan; **f** shows CD4 (orange) and PD-1 (cyan) overlaid to show that PD-1 mostly almost always colocalised with CD4 in follicular regions; **g** shows CD68 in magenta; **h** shows FOXP3 in green; **i** shows CD8 in yellow. **j** Summarises the methodology. Panels (k-l) demonstrate the process of observing spatial interactions: **k** shows FOXP3 (green) and CD8 (yellow) stains overlaid; **l** shows spatial “interactions” between cells scored as FOXP3^+^ (shown in red) and CD8^+^ (shown in yellow) are plotted as connections (shown in white) between cells occurring within 30 μm of each other

Stained sections were scanned with the Vectra 3.5 microscope (Akoya Biosciences). Initially, a low-resolution scan (10x) was performed to manually annotate the TMA core locations. Then, a multi-spectral image of each core was acquired at 20 × magnification (0.49 μm/pixel). Spectral unmixing [34] was performed using inForm 2.4 software (Akoya Biosciences). To separate the fluorophore signals, a spectral library was pre-built; the individual spectrum of each fluorophore, DAPI, and auto-fluorescence was acquired from single-plex controls. After unmixing, the images consisted of 6 channels, each containing the intensities of a different fluorophore, plus two channels for DAPI and auto-fluorescence ($2420\times 2420\times 8$ pixels). All prepared images (example in Fig. 1) are publicly available at http://dx.doi.org/10.17632/274xbhc5rx.3. Areas containing artefacts were manually excluded.

### Image analysis

#### Cell detection

Nuclear detection in these images was challenging, because of densely packed and overlapping cells. Therefore, label-free cell detection, as offered by inForm software, was insufficient. Instead, we trained a deep learning model on the DAPI channel using the “StarDist” method [35] (code available: https://github.com/mpicbg-csbd/stardist). The model was trained on 67,991 nuclear outline annotations, was fine-tuned using an additional validation set of 906 nuclei, and achieved average precision of 83% when tested on 883 unseen nuclei. The training, validation, and test sets were created for the purpose of this study by a trained non-expert, under supervision from a pathologist (R.B.). More details on the performance validation can be found in the supplementary material (Supplementary Material Sect. 3, Supplementary Fig. 6, and Supplementary Table 2). The nuclear annotations are publicly available (http://dx.doi.org/10.17632/nb46s9trx3.1). After nuclear detection, simulated membranes were grown around the nuclei by maximum 1.5 μm to represent whole cells (Supplementary Fig. 7) and measurements are taken of the median intensity for all stains and each cell compartment (nucleus, membrane).

#### Cell scoring

A single threshold was selected to mark positive cells for each stain across the entire dataset by observing the median stain intensity on the relevant compartment (nucleus for FOXP3 and membrane for all others). To select positivity thresholds, two annotators examined 10 multiplex images (Supplementary Material Sect. 4 and Supplementary Table 3), and the average threshold values were chosen. Cell density was subsequently measured for each cell phenotype of interest by dividing the number of positive cells by the tissue area.

When testing the prognostic significance of individual cell subsets, the total number of cells expressing a stain were observed to facilitate comparisons with prior FL studies. For example, when observing the cell subset that expressed CD68, all CD68^+^ cells were counted, regardless of the expression of other markers. In this way, the findings can be compared with previous FL studies that used single-plex assays to observe the prognostic value of CD68 stain [9–12].

#### Total immune infiltrate ratio

Additionally, the total immune infiltrate was measured as the number of cells expressing any of the CD4, FOXP3, CD8, CD68, or PD-1 markers. The immune infiltrate ratio was subsequently calculated by dividing the immune infiltrate cells by the number of all non-immune cells that expressed only DAPI. This ratio can be used to represent the extent of total immune infiltration in the microenvironment of FL.

#### Identifying CD21+ follicular dendritic meshwork areas

To quantify the extent of CD21^+^ follicular dendritic meshwork areas, manual annotations were drawn around them for all samples (Supplementary Fig. 8**),** under supervision of a pathologist. Subsequently, the area covered by these meshwork patterns was measured and expressed as a proportion of the total tissue area.

### Quantifying diversity of immune infiltrate

The next section introduces a method to objectively quantify the diversity of the non-neoplastic immune microenvironment. The cell types studied in the diversity analyses were not predetermined. Instead, all five different stains (CD4, CD8, CD68, FOXP3, PD-1) were observed for cell phenotyping. Each possible combination of stain expression was regarded as a distinct phenotype. As an example, CD4^+^CD8^−^CD68^−^FOXP3^+^PD-1^−^ was considered distinct from CD4^+^CD8^−^CD68^−^FOXP3^+^PD-1^+^. In this categorisation there exists a total of ${2}^{5} = 32$ stain positivity combinations, and each cell can only belong to a single phenotype. Cells expressing none of these markers were not included in the diversity analysis, as the aim was to assess the diversity of the non-neoplastic immune tumour microenvironment. Therefore, cells positive only for DAPI or only for CD21 were excluded as these could potentially include FL B-cell subsets.

#### Immune infiltrate diversity quantification

Immune infiltrate diversity was assessed by computing Shannon’s entropy for cell populations. Shannon’s entropy was calculated as:

1 $$ {\text{Entropy}} = - \mathop \sum \limits_{i}^{N} p_{i} \ln (p_{i} ) $$

where $N$ is the number of all possible cell phenotypes and ${p}_{i}$ the proportion of each phenotype $i$ in a sample.

#### Diversity of spatial interactions

The diversity of spatial interactions in each sample was additionally quantified by applying the HID methodology [27]. HID performs a pair-wise examination of cell types identified during cell scoring and counts their spatial interactions, i.e. their frequency of co-occurrence within a pre-specified distance (see Fig. 1k–l). Further implementation details can be found in Rose et al. [27]. The distance parameter was selected as 30 μm similar to the study of Tsakiroglou et al. [36], which represents a neighbourhood of 3–4 cells. A co-occurrence between each pair of phenotypes $i$ and $j$ was considered a unique type of spatial interaction. The proportion of all interactions belonging to this type ${p}_{i,j}$ could then be calculated. If we consider each type of interaction as a separate “species”, Shannon’s entropy diversity index for the distribution of interactions in a sample can be derived:

2 $$ {\text{Interaction}} \;{\text{entropy}} = - \mathop \sum \limits_{j = 1}^{N} \mathop \sum \limits_{i = j}^{N} p_{i,j} \ln \left( {p_{i,j} } \right) $$

In the current study all $N$ phenotypes that were observed in the samples across the entire dataset were assessed, while cells expressing only DAPI were ignored. Intuitively, interaction entropy quantifies the diversity of co-occurrences between immune cell subsets.

A summary of the methodology steps is given in Fig. 1j.

### Statistical analysis

**Fig. 2:**
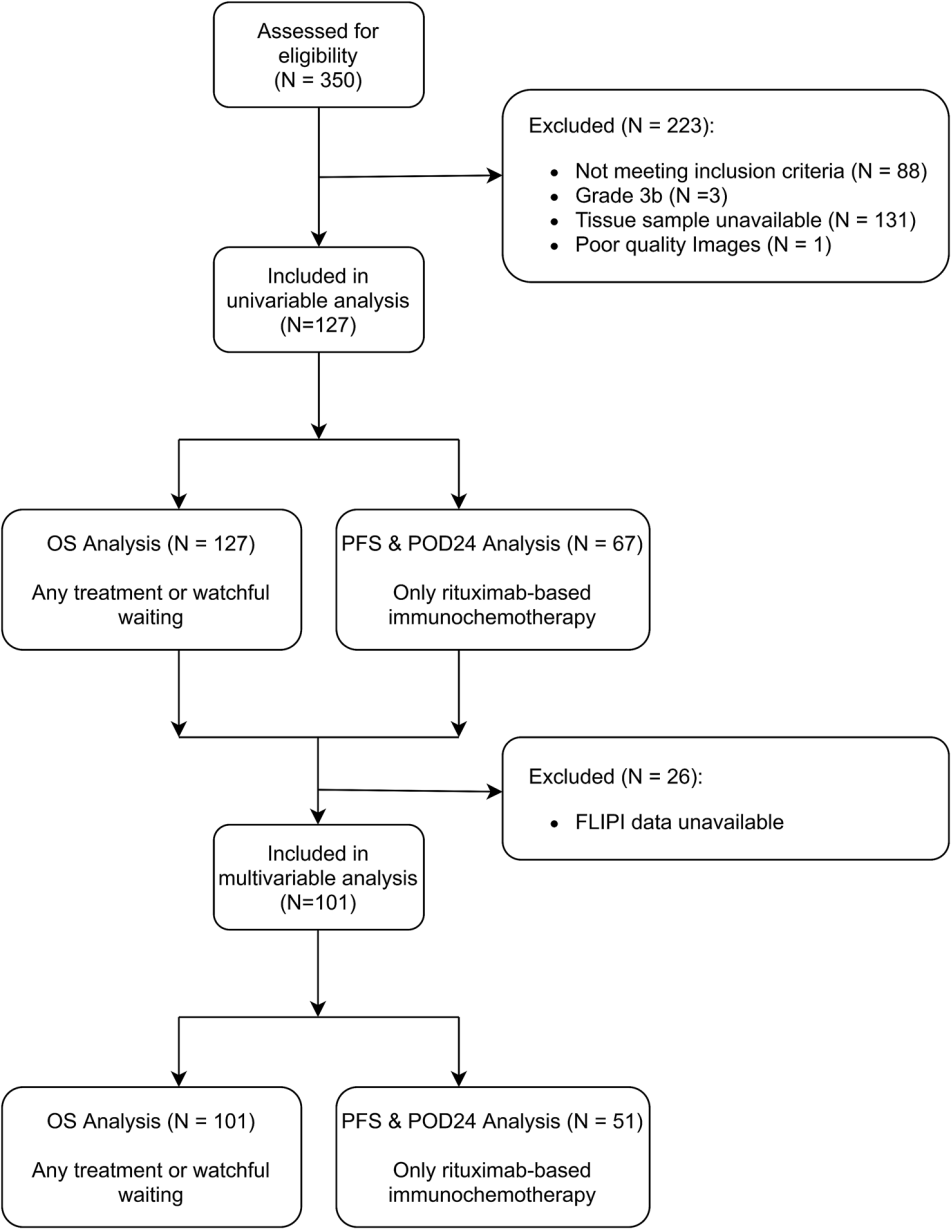
Flow chart of patients in the study. OS indicates overall survival; PFS progression-free survival and POD24 disease progression within 24 months of starting treatment

A patient flow chart for the analyses is presented in Fig. 2. Since cores were extracted in triplicate for each patient, the median feature value was used to represent the patient. Univariable analysis for OS and PFS was carried out using Cox regression models, where all features were treated as continuous variables. Multivariable analysis involved building Cox regression models to assess associations, independent of FLIPI. FLIPI was assessed as an ordinal score 0–5 [4]. FLIPI was selected for multivariable analyses as a state-of-the-art, widely validated prognostic index. We did not test histologic grade in multivariable analyses as its prognostic value for FL is limited and patients with grade 1–2 and 3a have similar survival rates [37–39].

Kaplan–Meier analysis for the diversity features was carried out by dichotomising the variables at the optimal cut point. This cut point was selected to maximise the difference in survival characteristics between the groups, and an adjusted p value was provided to account for bias using the Contal and O’Quigley method [40]. The findcut implementation in SAS 9.4 was used for this optimal cut-point selection [41]. Confidence intervals were calculated in Kaplan–Meier analysis using Greenwood’s formula [42].

Differences between POD24-positive and POD24-negative groups were tested using the nonparametric Mann–Whitney U test. Univariable and multivariable logistic regression was also applied for POD24 prediction. All patients included in POD24 analyses had at least 24 months of follow-up.

Significance was assessed at a level *α* = 0.05, and the Bonferroni correction was applied to account for multiple hypothesis testing. Statistical tests were performed using the lifelines v.0.14.6, statsmodels v.0.10.1, and scipy v.1.3.1 libraries in Python.

## Results

### Cohort characteristics

The 127 FL patients were initially treated with rituximab-based therapy (52%), radiotherapy (28%), or active surveillance (20%). The 3- and 5-year OS rates were 97.7% (95% CI: 92.67, 99.22) and 94.46% (95% CI: 88.51, 97.14). For POD24 analysis, 67 patients had a minimum of 2-year follow-up, and 14 had an observed progression event within 24 months of the initiation of immuno-chemotherapy (20.3%). POD24 was an indicator of unfavourable OS (*p* = 0.001) and PFS (*p* < 0.001) (Supplementary Fig. 9). FLIPI data were available at diagnosis for 101 patients, of which 51 were treated with rituximab-based regimens. FLIPI takes into account age, stage, haemoglobin levels, lactate dehydrogenase (LDH) levels, and number of nodal site involvement. The distribution of FLIPI index risk (low: 44%, intermediate: 33%, high: 23%) is similar to that reported by others [5].

Supplementary Table 4 summarises all patient characteristics at diagnosis, and Supplementary Material Sect. 8 describes the prognostic value of clinical characteristics commonly used to assess patient risk (e.g. FLIPI) in univariable Cox regression analysis (Supplementary Table 5).

### Distribution of immune cell densities and diversity metrics

Supplementary Table 6 provides the median, inter-quartile range, and intra-patient coefficient of variation (CoV) of cell populations and diversity metrics in the 127-patient cohort. The CoV measures intra-patient heterogeneity between different TMA cores for the same patient. The CoV of the diversity metrics was very low (7.7% and 8.3% for interaction and phenotype entropy, respectively), indicating that diversity in FL could be accurately measured by use of triplicate core samples.

### Cell population densities were not prognostic in multivariable analysis

In univariable Cox regression for OS, only the density of macrophages (HR = 0.99, 95% CI 0.98, 1.0) was significant after the Bonferroni correction for multiple comparisons (Table 1). However, in univariable Cox regression for PFS (Table 1), and all multivariable analyses (Table 2), none of the cell population densities were statistically significant.

**Table 1:** Univariable survival analysis for features derived from the tumour microenvironment

	Univariable Analysis	Cox PH Univariable OS All Patients, *N* = 127, 27 Events		Cox PH Univariable PFS Rituximab Patients, *N* = 67, 39 Events	
		HR (95% CI)	P^*^	HR (95% CI)	P^*^
Cell density, cells/mm^2^	CD4^+^CD68^−^ T-helper cells	1 (1, 1)	0.264	1 (1, 1)	0.160
	CD4^+^FOXP3^+^ T-regs	0.96 (0.92, 0.99)	0.023	0.97 (0.95, 1)	0.022
	CD8^+^ T-cells	0.99 (0.99, 1)	0.055	1 (0.99, 1)	0.211
	CD68^+^ cells	0.99 (0.98, 1)	0.002	0.99 (0.99, 1)	0.010
	CD4^+^CD68^−^PD-1^+^	0.99 (0.98, 1.01)	0.278	1 (0.99, 1.01)	0.467
	CD8^+^PD-1^+^	0.97 (0.94, 1)	0.084	0.99 (0.97, 1.01)	0.253
Cell ratio	Immune infiltrate ratio†	0.21 (0.05, 0.92)	0.039	0.25 (0.08, 0.82)	0.023
% Positive area	CD21^+^ dendritic meshwork area	1.65 (0.31, 8.8)	0.556	1.35 (0.41, 4.48)	0.626
Diversity, natural digits	Phenotype entropy	0.22 (0.07, 0.64)	0.006	0.69 (0.3, 1.61)	0.393
	Interaction entropy	0.47 (0.27, 0.82)	0.007	0.81 (0.52, 1.27)	0.359

*HR* hazard ratio; *CI* confidence intervals; *PH* proportional hazards; *OS* overall survival; *PFS* progression-free survival. *The log rank test *p* value examines whether the null hypothesis of no effect (H_0_: HR = 1) can be rejected. †Immune infiltrate ratio is calculated as the total immune cells (positive for any marker) divided by the number of cells that expressed only DAPI. *P* values $\le$ 0.05 are shown in bold. All features were assessed as continuous variables. *P* values $\le$ 0.005 remain significant after the Bonferroni correction for multiple hypothesis testing

**Table 2:** Multivariable survival analysis for features derived from the tumour microenvironment

	Multivariable Models with FLIPI	Cox PH Multivariable OS		Cox PH Multivariable PFS	
		All Patients, *N* = 101, 20 events		Rituximab Patients, *N* = 51, 29 events	
		HR (95% CI)	P^*^	HR (95% CI)	P^*^
Cell density, cells/mm^2^	CD4^+^CD68^−^ T-helper cells	0.872	0.872	1 (1, 1)	0.158
	CD4^+^FOXP3^+^ T-regs	0.96 (0.92, 1)	0.066	0.98 (0.95, 1)	0.109
	CD8^+^ T-cells	1 (0.99, 1)	0.315	1 (0.99, 1)	0.561
	CD68^+^ cells	0.99 (0.98, 1)	**0.013**	0.99 (0.99, 1)	**0.046**
	CD4^+^CD68^−^PD-1^+^	1 (0.98, 1.01)	0.478	1 (0.99, 1.01)	0.907
	CD8^+^PD-1^+^	0.97 (0.94, 1.01)	0.137	1 (0.98, 1.01)	0.613
Cell Ratio	Immune infiltrate ratio^†^	0.37 (0.07, 2)	0.247	0.35 (0.09, 1.37)	0.131
% Positive Area	CD21^+^ dendritic meshwork area	0.4 (0.09, 1.69)	0.212	1.08 (0.25, 4.79)	0.915
Diversity, natural digits	Phenotype entropy	0.19 (0.06, 0.65)	**0.008**	0.85 (0.31, 2.31)	0.750
	Interaction entropy	0.39 (0.2, 0.75)	**0.005**	0.9 (0.53, 1.53)	0.700

Only subset of patients with available FLIPI data at diagnosis is included. *HR* hazard ratio; *CI* confidence intervals; *PH* proportional hazards; *OS* overall survival; *PFS* progression free survival. *The log rank test *p* value examines whether the null hypothesis of no effect (H0: HR = 1) can be rejected. Features are assessed as continuous variables. †Immune infiltrate ratio is calculated as the total immune cells (positive for any marker) divided by the number of cells that expressed only DAPI. *P* values $\le$ 0.05 are shown in bold. *P* values $\le$ 0.005 remain significant after the Bonferroni correction for multiple hypothesis testing

### Increased total immune infiltrate was seen in patients without POD24

**Fig. 3:**
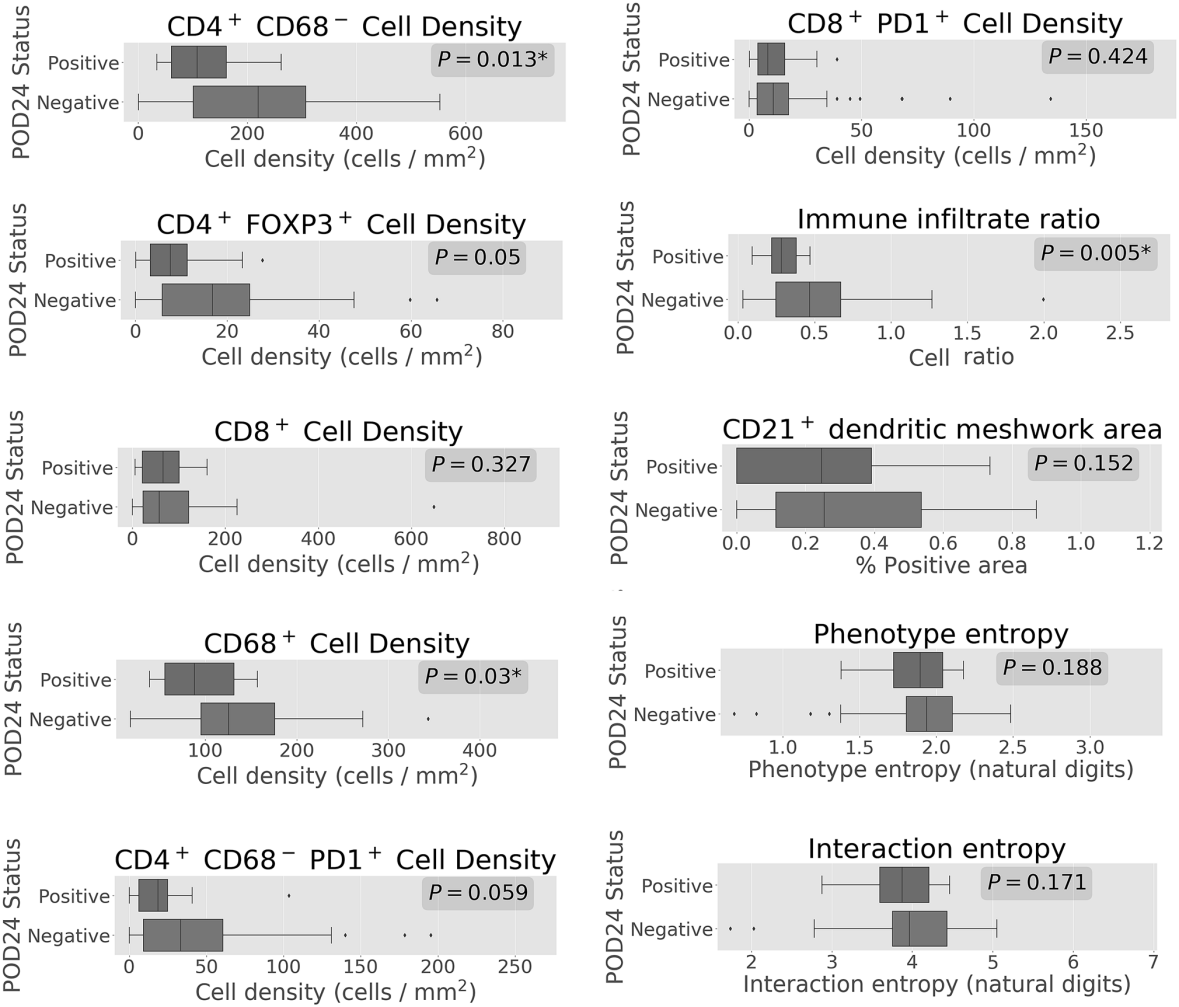
Differences in immune cell density and tumour microenvironment diversity between POD24-positive and POD24-negative subgroups. Cell densities are shown in cells/mm^2^, while Shannon entropy is presented in natural bit units

Higher density of several immune subsets was seen in the group without POD24 (Fig. 3), and an increased total immune infiltrate ratio was significantly favourable for POD24 after Bonferroni adjustments. However, none of features remained significantly correlated with POD24 in univariable or multivariable logistic regression analyses (Table 3).

**Table 3:** Logistic regression for POD24 prediction in the subset treated with rituximab containing regimens

	Logistic Regression for POD24	Univariable		Multivariable with FLIPI	
		Rituximab patients, *N* = 67 [14 events]		Rituximab patients, *N* = 51 [8 events]	
		OR (95% CI)	P^*^	OR (95% CI)	P^*^
Cell density, cells/mm^2^	CD4^+^CD68^−^ T-helper cells	0.99 (0.99, 1)	**0.027**	0.99 (0.98, 1)	**0.034**
	CD4^+^FOXP3^+^ T-regs	0.95 (0.9, 1)	0.066	0.95 (0.88, 1.02)	0.132
	CD8^+^ T-cells	1 (0.99, 1.01)	0.465	1 (0.99, 1.01)	0.638
	CD68^+^ cells	0.99 (0.98, 1)	0.051	0.98 (0.97, 1)	0.063
	CD4^+^CD68^−^PD-1^+^	0.98 (0.96, 1.01)	0.116	0.96 (0.92, 1.01)	0.100
	CD8^+^PD-1^+^	0.98 (0.95, 1.02)	0.389	0.97 (0.92, 1.04)	0.410
Cell ratio	Immune infiltrate ratio^†^	0.02 (0, 0.48)	**0.017**	0.01 (0, 1.23)	0.060
% Positive area	CD21^+^ dendritic meshwork area	0.28 (0.02, 3.65)	0.334	0.06 (0, 3.14)	0.166
Diversity, natural digits	Phenotype entropy	0.73 (0.13, 4.07)	0.718	0.64 (0.08, 5.07)	0.669
	Interaction entropy	0.82 (0.33, 2.02)	0.665	0.75 (0.25, 2.27)	0.610

POD24 indicates disease progression within 24 months of starting treatment; OR indicates odds ratio. Only subset of patients with available FLIPI data at diagnosis is included in multivariable analysis and features treated as continuous variables. *The log rank test *p* value examines whether the null hypothesis of no effect (H0: odds ratio = 1) can be rejected. †Immune infiltrate ratio is calculated as the total immune cells (positive for any marker) divided by the number of cells that expressed only DAPI. Features are assessed as continuous variables. *P* values $\le$ 0.05 are shown in bold. *P* values $\le$ 0.005 would remain significant after the Bonferroni correction for multiple hypothesis testing

### Immune infiltrate diversity analysis

Increased diversity of cell types (HR = 0.22, 95% CI 0.07, 0.64) and diversity of spatial interactions (HR = 0.47, 95% CI 0.27, 0.82) were favourable for OS in univariable Cox regression analysis (*N* = 127, Table 1). Furthermore, in multivariable Cox regression analysis (*N* = 101, Table 2), the diversity of spatial interactions was favourable for OS (HR = 0.39, 95% CI 0.20, 0.75) and remained significant after Bonferroni correction. Therefore, the immune diversity biomarker offers prognostic value which is independent of FLIPI.

**Fig. 4:**
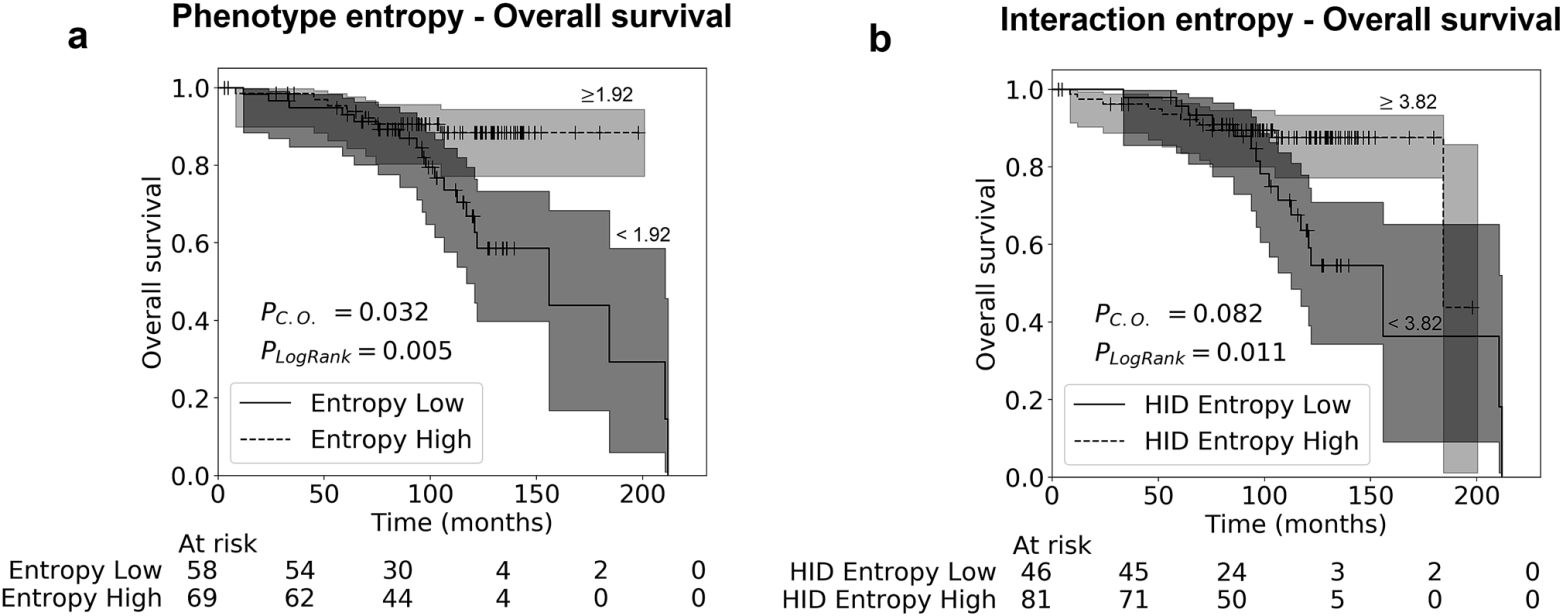
Kaplan–Meier survival analysis. Analysis shown for OS, where patients have been split into two groups based on the optimal cut points. Cut points were selected using the Contal & O’Quigley test [38] method. P_Log Rank_: significance for the log rank test and P_C.O._: significance for the Contal & O’Quigley test [38], adjusted for the fact that the optimal cut point has been selected to maximise separation of patient groups. Shaded areas represent 95% confidence intervals [42]. **a** Effect of Shannon phenotype entropy (diversity) on OS; **b** effect of HID spatial “interaction” entropy (diversity) on OS

Kaplan–Meier analysis showed a trend towards increased diversities being favourable for OS (Fig. 4), when stratified at the optimal cut-off. The optimal cut-off was selected using the Contal & O’ Quigley [41] method, where all possible cut-offs are tested and the p value is adjusted to account for the bias of multiple testing. Stratification of OS based on the diversity of phenotypes was significant (Contal & O’ Quigley [41] adjusted *p* = 0.032), assigning 45.6% of patients to the poor prognostic group.

Even though the diversity biomarkers predicted favourable OS, no associations were observed with PFS (Tables 1, 2) and POD24 (Table 3, Fig. 3).

## Discussion

This study introduced a 6-plex immunofluorescence protocol for concurrent observation of immune subsets and an image analysis pipeline to accurately detect cell types and objectively measure tumour microenvironment diversity. This new approach provides a versatile and adaptable platform that could be extended to other tumour types. The proposed pipeline benefits from precise marker localisation as well as conservation of valuable tissue material through multiplexing. The improved accuracy and reliability of quantitative immunofluorescence compared to conventional immunohistochemistry, and its cost-effectiveness compared to in situ hybridisation, provide scope and rationale for wider clinical adoption.

Developing baseline prognostic biomarkers for risk stratification is a major area of research in FL, driven by an urgent need to develop effective therapies capable of improving the outcomes of high-risk disease. Using this pipeline, we report that increased diversity of immune infiltrate populations and interactions in FL is potential biomarkers of favourable OS. Diversity was quantified through a novel approach using Shannon’s entropy, a metric describing species biodiversity in ecological sciences. The diversity of spatial interactions remained significant after Bonferroni correction for multiple comparisons in multivariable analysis of OS. Therefore, this biomarker could improve risk stratification, offering additional prognostic value when combined with FLIPI assessment. The diversity biomarkers also outperformed simple cell density measurements. Indeed, none of the immune infiltrate cell densities remained significantly associated with survival endpoints in multivariable analysis (Table 2), similar to results reported by others [14] for rituximab-treated patients. This evidence supports applicability of the diversity biomarker for risk stratification in FL.

Survival analysis (Tables 1, 2, 3) in this study treated all variables as continuous, to avoid the loss of information from arbitrary dichotomisation [43]. However, since dichotomisation is sometimes required for clinical decision-making, we also carried out Kaplan–Meier analysis by selecting a cut-off to split the patients in two groups. Clinical studies often select the median or quartiles as the cut-off, even though is no underlying statistical reasoning for this selection [44]. We adopted the Contal and O’Quigley approach [40], as it provides a non-arbitrary cut-off selection and supplies a corrected *p* value, taking into account the inflated type-I error that may result from testing multiple cut-offs. Further validation of this cut-off in additional cohorts would be beneficial. However, since we performed Cox regression analyses without dichotomising the variables, the reported prognostic value does not rely upon a specific cut-off selection.

Previous studies investigating tumour immune microenvironment diversity in other types of cancer have demonstrated the importance of diversity in T-cell populations, as measured by T-cell receptor (TCR) next-generation (NGS) sequencing, in a way that is agnostic to the types of T-cells that are quantified [45]. Increased TCR diversity has been associated with improved clinical benefit in metastatic melanoma [46] and favourable overall survival in metastatic breast cancer [47]. Furthermore, clonal TCR diversity has been shown to increase after immunotherapy treatments (e.g. cryo-immunotherapy for breast cancer [46] and Sipuleucel-T immunotherapy for prostate cancer [48]) and is investigated as a potential endpoint for response to therapy [47]. A diverse T-cell repertoire is thought to increase the likelihood that a useful anti-tumour T-cell population is present [46], leading to favourable outcomes. In this study we expand the concept of diversity to include T-cells and macrophages and propose that a diverse repertoire of immune cells in the microenvironment of FL could similarly increase the likelihood of relevant anti-tumour pathways being active.

In this study, CD68^+^ macrophages were significantly correlated with favourable OS in univariable analysis. A favourable trend of increased CD68^+^ density was observed for PFS and POD24. This effect could be attributed to one of the mechanisms of action of the anti-CD20 rituximab treatment, whose immune-mobilising effects include the induction of antibody-dependent cell phagocytosis [49]. Consequently, cells coated with rituximab are recognised by macrophages as targets and killed [50]. The favourable effect of macrophages has been previously demonstrated in a rituximab-treated cohort [10]. However, this effect depends strongly on the type of treatment, as in cohorts treated without rituximab [9, 11] increased numbers of tumour-associated macrophages correlated with unfavourable outcome.

To ensure reproducibility of results, we quantitatively validate the staining assay and cell detection algorithms and share publicly the image dataset [28]. The FL cohort included treatment pathways and prognostic outcomes reflective of current modern practice. The TMA technology employed is equivalent to whole section assessments in lymphomas [51], enabling rapid processing of large number of samples. Furthermore, the diversity metrics demonstrated low intra-patient heterogeneity (CoV = 7.7–8.3%), indicating robustness when assessed using triplicate TMA core samples.

A limitation of this study is the use of a single cut-off to score positive and negative cells for each stain. Robust cut-offs were selected by two different users of the computer-assisted scoring system. However, this approach may sometimes underperform because of the inherent variation of staining intensities in positive cells. Notably, in FL two functionally different PD-1^+^ cell phenotypes have been observed [20], characterised by different levels of PD-1 expression: PD-1^+^_high_ T follicular helper cells found inside the follicles actively support FL B-cell growth, while the PD-1^+^_low_ cells found outside the follicles represent exhausted T-cells. The PD-1^+^ T-helper cells found within the follicles are also known to express CD4 less strongly (30.7% lower CD4 intensity) compared to other CD4^+^ cells in the interfollicular areas [52]. The present study attempted to select single cut-offs able to pick up both the weakly and strongly positive cells. Use of multiple cut-offs was avoided as nuanced intensity variations can be challenging to capture using manual gating in a reproducible manner. Future work could adopt automated clustering [53] of cells based on their intensities or rely on additional functional markers in the multiplex panel (e.g. TIM3 for exhausted phenotypes or CXCR5 for T follicular helper cells [20]) to differentiate between PD-1 subsets.

Cox regression for PFS and logistic regression for POD24 in the rituximab-treated subset did not demonstrate significant prognostic value for any of the immune infiltrate biomarkers. However, the limited size and variable treatment increase the risk of false-negative results. Therefore, the effect of tumour microenvironment diversity on early relapse merits further investigation before it could be ruled out. A significantly lower total immune infiltrate ratio was observed in patients that had POD24, as seen in Fig. 3. Even though the causality underpinning this observation is not well understood, the total immune infiltrate ratio may be further investigated as an indicator of POD24 that can be calculated at baseline, before treatment has started. Additional markers of B-cells and particularly of FL B-cells would be beneficial for a meaningful study of the “DAPI only” cells. The lack of a FL tumour marker is a limitation of this study, which prioritised the analysis of the non-neoplastic, immune microenvironment. By assessing 7 markers (DAPI, CD4, CD8, CD68, FOXP3, CD21, PD-1), we have reached the limit in number of markers permitted by the multiplex staining and imaging platform used in this study.

In summary, automated assessment of immune infiltrate and its diversity, based on multiplex immunofluorescence, warrants further exploration for prognostic biomarker development in FL. Future work may involve validation of diversity measurements using orthogonal assays, such as gene expression profiling. This pipeline is ready to be tested in larger series, with the potential to significantly improve risk stratification and treatment adaptation for high-risk FL in the future.

### Supplementary Information

Below is the link to the electronic supplementary material.

Supplementary file1 (PDF 1362 kb)

## Data Availability

Python code is available upon request from the corresponding authors.

## Data availability

The multiplex image dataset and annotations for nuclear segmentation are available at 10.17632/274xbhc5rx.3. The multiplex protocol is available at 10.17504/protocols.io.49ygz7w .
