# Supplementary material for: Immune infiltrate diversity confers a good prognosis in follicular lymphoma
Source: Cancer Immunol Immunother. 2021 Apr 30;70(12):3573–85. doi: 10.1007/s00262-021-02945-0 (PMC8571143; doi:10.1007/s00262-021-02945-0)
Supplement: Supplementary file 1 — Supplementary file1 (PDF 1362 kb) [file 262_2021_2945_MOESM1_ESM.pdf]

1. **Comparison of overall survival in patients with and without available samples**

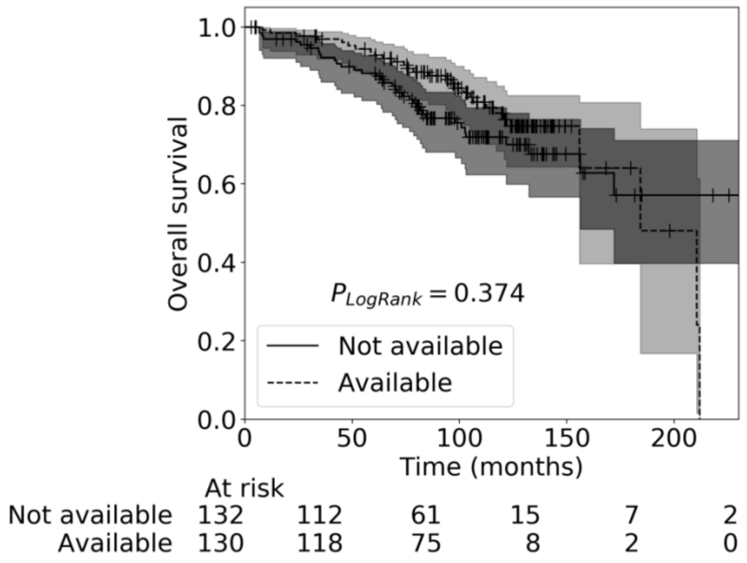

Fig. 1 Kaplan-Meier analysis of overall survival (months) for all requested samples in the FL cohort (N=262), stratified for tumour sample availability. Censored data are marked with crosses (log rank test p=0.374).

2. Validation of multi-plex immunofluorescence protocol

Table 1 Antibodies, titrations and fluorophores in the multi-plex immunofluorescence protocol. The order reflects the order the antibodies are applied on the tissue.

Order	Antibody	Dilution	Provider	Opal detection
1	Anti-CD4 (SP35) Rabbit Monoclonal Primary Antibody	Pre-diluted	Roche, Switzerland	Opal 620
2	Anti-CD68 antibody mouse monoclonal [KP1] to CD68	1:40	Abcam, UK	Opal 650
3	Monoclonal Mouse Anti-Human CD8 Clone C8/144B	1:450	Agilent, Denmark	Opal 540
4	CD21 (2G9) Mouse Monoclonal Antibody	1:25	Cell Marque, USA	Opal 570
5	Anti-FOXP3 antibody mouse monoclonal [236A/E7]	1:60	Abcam, UK	Opal 520
6	Anti-PD-1 antibody [NAT105] (ab52587) Mouse monoclonal	1:150	Abcam, UK	Opal 690

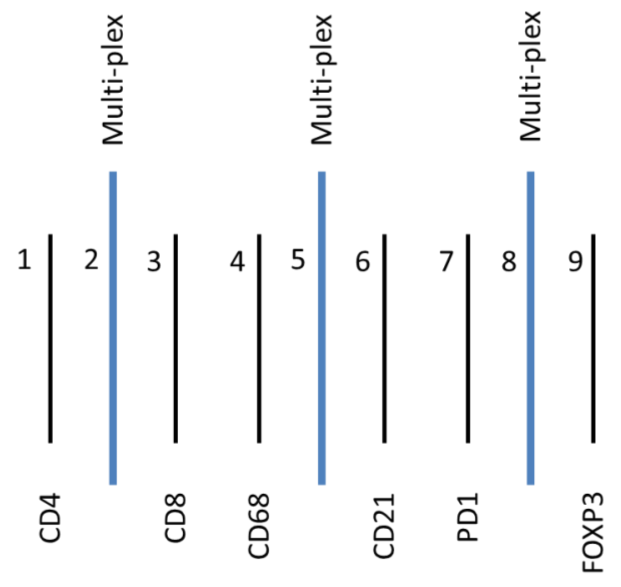

Fig. 2 Sequential TMA sections setup for multi-plex experiment validation. Each single-plex assay is compared to an adjacent multi-plex assay.

Experimental assay development and validation was performed using sequential sections from a FFPE TMA constructed from 44 FL cases retrospectively collected from The Christie archives. These patients were diagnosed in the 1980-1990s and treated using historical protocols.

A detailed version of the multi-plex immunofluorescent experiment can be found in Tsakiroglou et al. [33]. To establish agreement between single-plex and multi-plex assays we stained pairs of 4μm thick, sequential sections from a follicular lymphoma TMA block. In each pair one section was stained using the multi-plex and the other using a single-plex protocol (Supplementary Fig. 2). DAPI was added in both the single-plex and multi-plex experiments to quantify the whole tissue area in each core. Slides were scanned multispectrally on the Vectra microscope (Akoya Biosciences, software version 3.5) at 20x magnification, and the exposure times were set according to the observed signal strength of each filter. In the case of the single-plex experiments, exposure times were adjusted only for the relevant filters, while for the rest the default settings

were applied; 40 ms for the overview and 150 ms for the multi-spectral scan. A spectral library was built and spectral unmixing of all sections was carried out in inForm 2.4 software (Akoya Biosciences).

Image analysis was subsequently performed in HALO software (Indica Labs, Albuquerque, NM, USA). Using the Multi-plex Fluorescent Area Quantification module, automated thresholding of pixel intensities in each channel identified the percentage of stained area. A demonstration of automated area quantification is shown in Supplementary Fig. 3. This algorithm requires the user to specify minimum true signal intensity. These settings for the single-plex and multi-plex sequential sections were chosen by the same user, leaving a "washout" period of 3 days between them. Cores with artefacts, such as bubbles and blood vessels were excluded from the analysis. In some cases, cores would be missing from one of the two sequential sections (tissue was broken or torn), and so these were excluded as well.

Comparisons between single-plex and multi-plex experiments demonstrated satisfactory linear correlations as shown in Supplementary Fig. 4. For most markers, slightly lower staining expression was observed in the multi-plex compared to the single-plex experiments. This was not observed for CD4, the first antibody placed on the tissue. Lower expression may derive from incomplete stripping in between staining cycles, which may lead to steric obstruction and slightly decreased antibody binding. This effect was however not significant, as seen in Bland-Altman plots (Supplementary Fig. 5). The mean difference of the two experiments was usually close to zero, with ≥95% of data points lying within the limits of agreement (mean ± 1.96 standard deviation) for all markers.

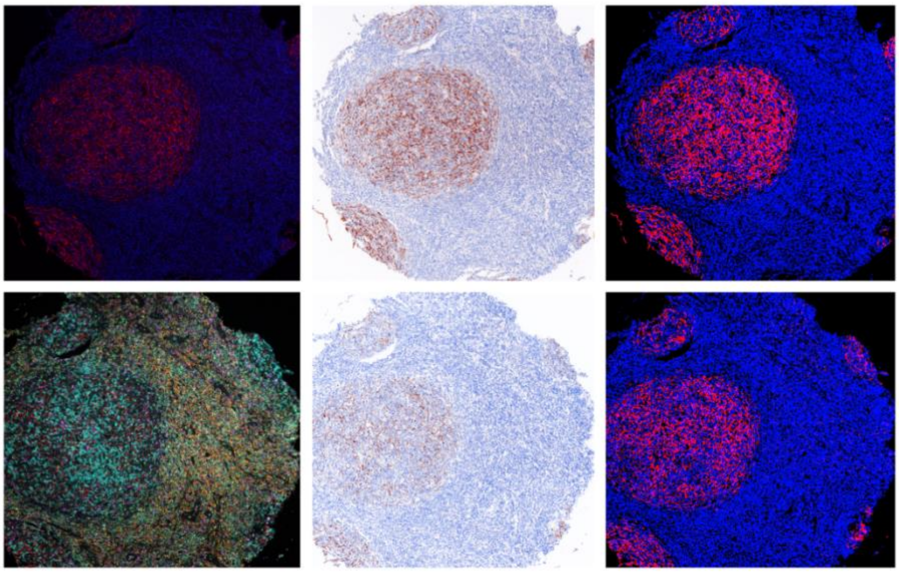

Fig. 3 Area quantification in HALO for the CD21 antibody (570 fluorophore). Top row: single-plex. Bottom row: multi-plex. Left: Unmixed composite image. Middle: simulated chromogenic view (inForm 2.4) for CD21. Right: Positive area quantification (HALO) where CD21 is rendered in red and DAPI in blue.

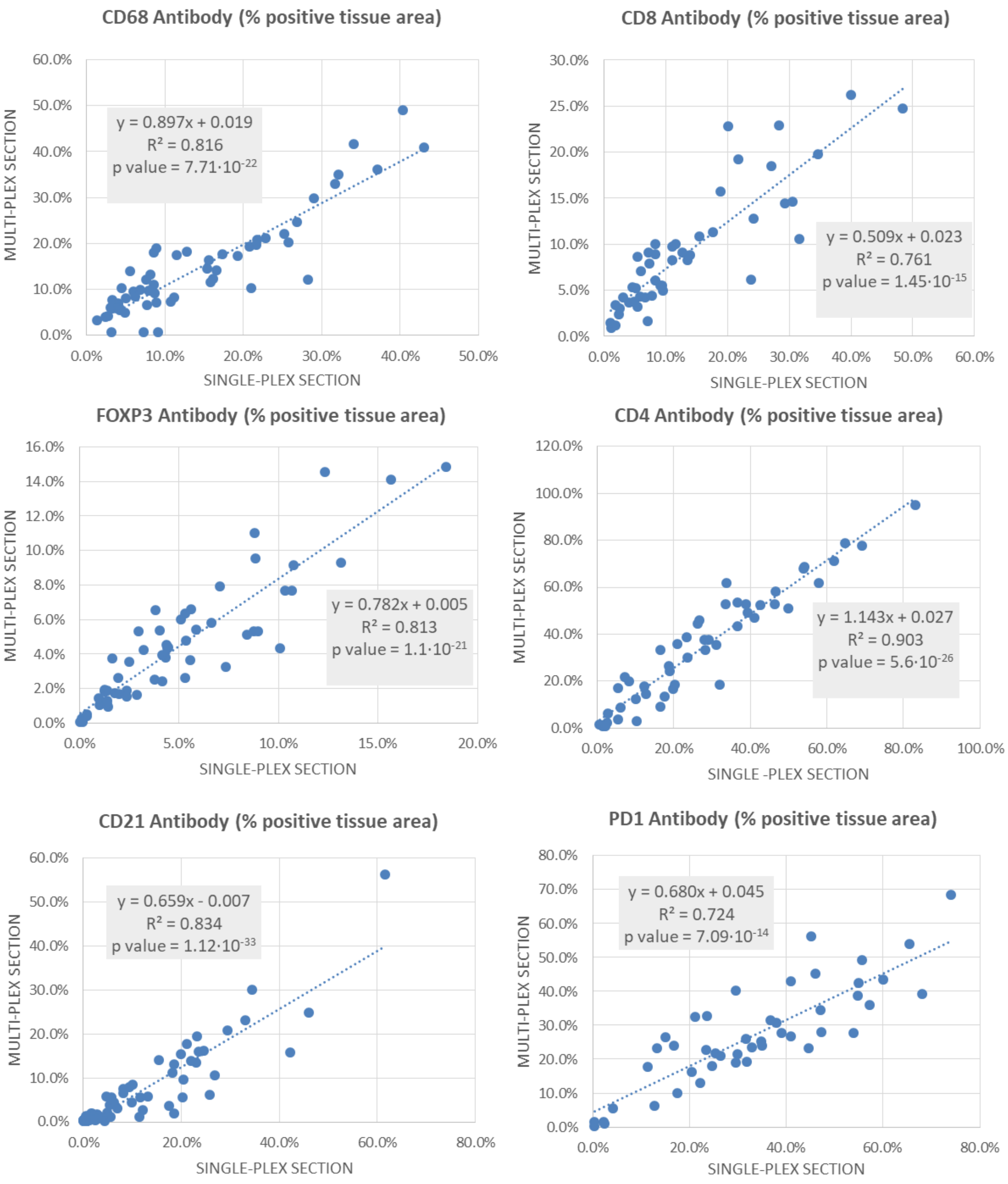

Fig. 4 Comparison of % tissue area stained by each marker in two sequential 4µm TMA sections, a multi-plex and a single-plex. The single-plex was also stained with DAPI and both sections were scanned multi-spectrally at 20x and unmixed with the same spectral library. Each point represents a TMA core.

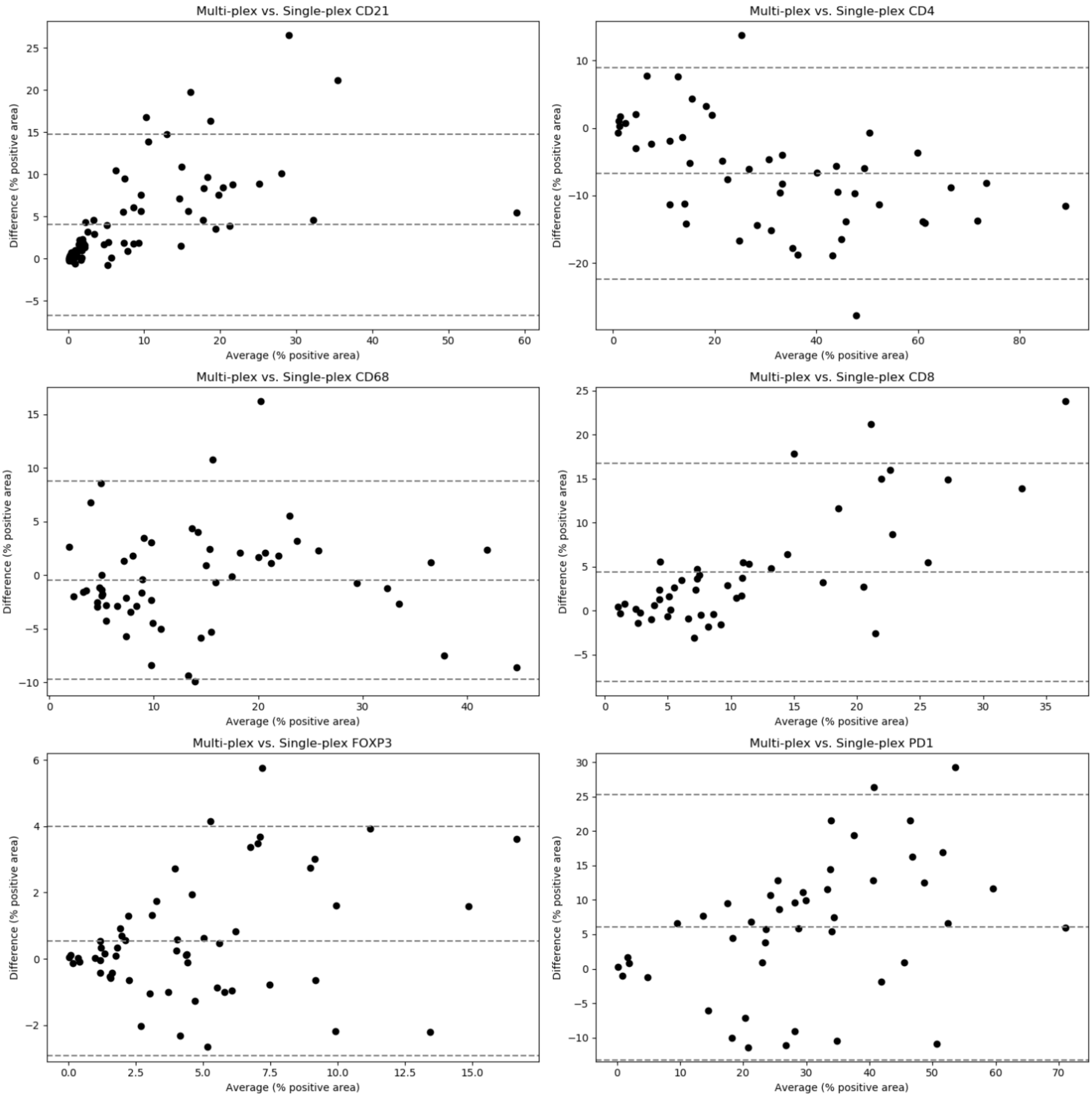

Fig. 3 Bland-Altman plot comparisons between single-plex and multi-plex immunofluorescent assays for each antibody. Antibody expression is measured as a percentage of the positively stained tissue area. Each point represents a TMA core. The dotted lines represent the limits of agreement (± 1.96 standard deviation of difference).

3. Cell detection

To assess segmentation performance, we considered the average precision $AP = \frac{TP}{TP+FP+FN}$, where true positive (TP) predictions are defined as predicted nuclei, for whom exist ground truth (GT) nuclei with sufficient overlap. Overlap was measured as intersection over union (IoU) > 30%. False positive (FP) were the unmatched predicted nuclei, while false negative (FN) were the unmatched ground truth nuclei. There were 3 ROI (883 nuclei) in the test set, 3 ROI (906 nuclei) in the validation set and 35 ROI (67991 nuclei) in the training set. The average precision for the testing set of nuclei was AP = 0.827. The worst image in the testing set is presented in Supplementary Fig. 6 (AP=0.733) and in Supplementary Table 2 the AP for different threshold of the IoU is given for the test set.

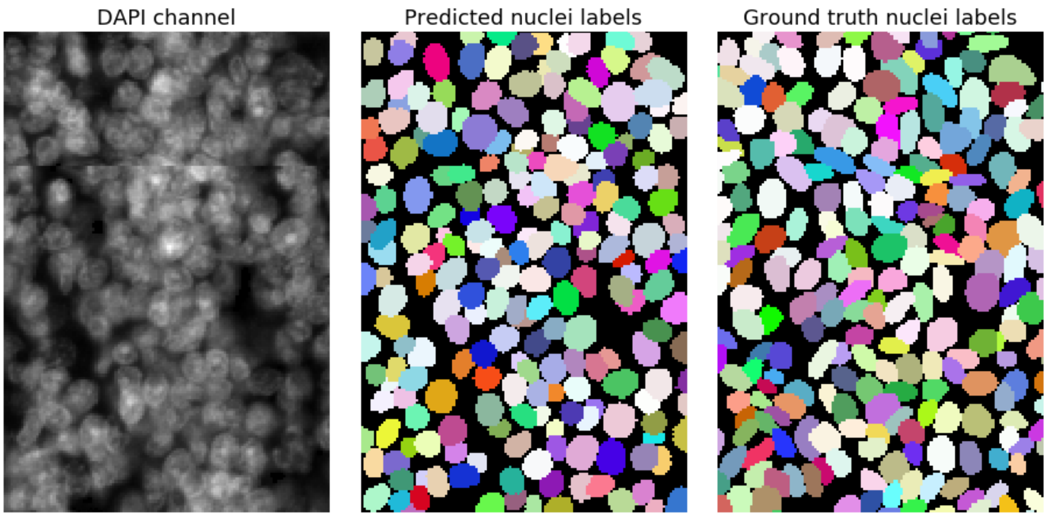

Fig. 4 Worse performing image in test set for nuclear segmentation (AP=0.733). The amount of overlap between nuclei is challenging even for human annotators.

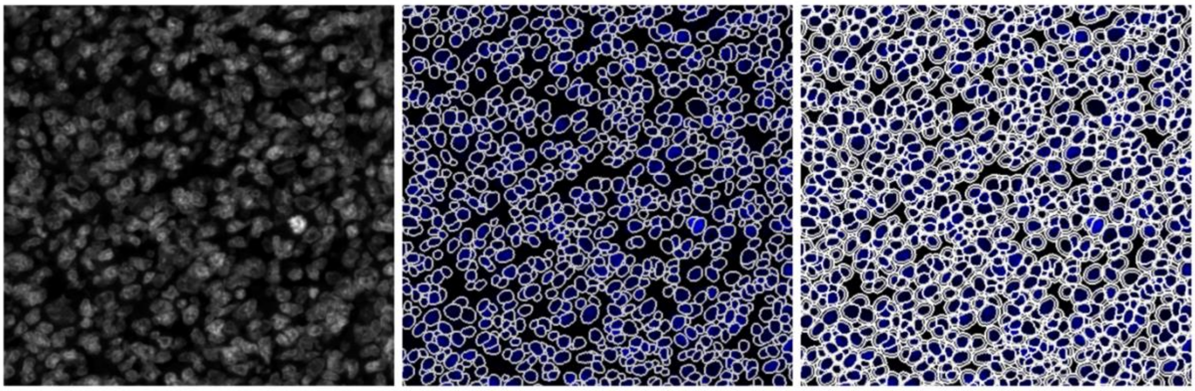

Fig. 5 Growing membranes around detected nuclei.

After nuclear segmentation, simulated membranes are grown around the nuclei by maximum 1.5 µm to represent whole cells (see Supplementary Fig. 7) and measurements are taken of the median intensity for all stains and each cell compartment (nucleus, membrane). All images in the dataset were manual examined and areas that presented artefacts because of folded tissue, bubbles or blood vessels were excluded from further analysis.

Table 2 Segmentation performance in the test set for different thresholds of the intersection over union (IoU) parameter. The test set included 3 ROI with 883 nuclei. AP: Average precision.

IoU threshold	0.1	0.2	0.3	0.4	0.5	0.6	0.7	0.8	0.9
AP	0.915	0.866	0.827	0.726	0.603	0.494	0.341	0.157	0.013

4. Positive cell scoring

A validation set of 10 images, each containing a whole core, was selected to determine a positivity cut-off for each stain, based on the median stain intensity of the relevant compartment (nuclear for FOXP3 and membrane for all the rest). The method used to determine the optimal value of the positivity cut-offs was as follows; first, intensity scaling onto a consistent colour map across all images was carried out for each stain so that equal intensity levels were represented by equal brightness. A cut-off threshold was selected per image core and stain by two independent annotators (a non-expert [A.M.T] and a trainee pathologist [M. D.]) to separate positive from negative cells. Agreement between the two annotators is shown in Supplementary Table 3. A single threshold was then selected as a positivity cut-off per stain by averaging all thresholds selected for the images in the validation set by both annotators.

Agreement was assessed by using the thresholds to classify the cells as positive or negative and calculating the f_1 score (harmonic mean of precision and recall) between the labels generated by different annotators. The fact that a single threshold across all images was mostly adequate to separate positive from negative cells ($0.68 \leq f_1$ score ≤ 0.92) indicates low staining variation across different patients and TMA blocks. These single cut-off thresholds were finally applied to phenotype cells in the entire data set.

Table 3 Agreement for cell labels generated by selecting a positivity cut-off per image in the validation set. Agreement is calculated as the $\boldsymbol{f_1}$score, representing the harmonic mean of precision and recall for the binary classification task of assigning a cell as positive or negative for each stain.

**FOXP3**	Annotator 1	Trainee pathologist	Single threshold
Annotator 1	-	0.83	0.92
Trainee pathologist		-	0.90
Single threshold			-
**CD8**	Annotator 1	Trainee pathologist	Single threshold
Annotator 1	-	0.72	0.86
Trainee pathologist		-	0.85
Single threshold			-
**CD4**	Annotator 1	Trainee pathologist	Single threshold
Annotator 1	-	0.88	0.87
Trainee pathologist		-	0.88
Single threshold			-
**CD68**	Annotator 1	Trainee pathologist	Single threshold
Annotator 1	-	0.49	0.76
Trainee pathologist		-	0.68
Single threshold			-
**PD-1**	Annotator 1	Trainee pathologist	Single threshold
Annotator 1	-	0.69	0.76
Trainee pathologist		-	0.86
Single threshold			-

This method was applied to identify cells positive for CD4, FOXP3, CD8, CD68 and PD-1. This approach was not adopted for CD21, as the staining pattern of $CD21^+$ cells followed a non-convex meshwork pattern which would be challenging to simulate accurately by simply growing simulated membranes around the nuclei.

5. Identifying $CD21^+$ meshwork pattern areas

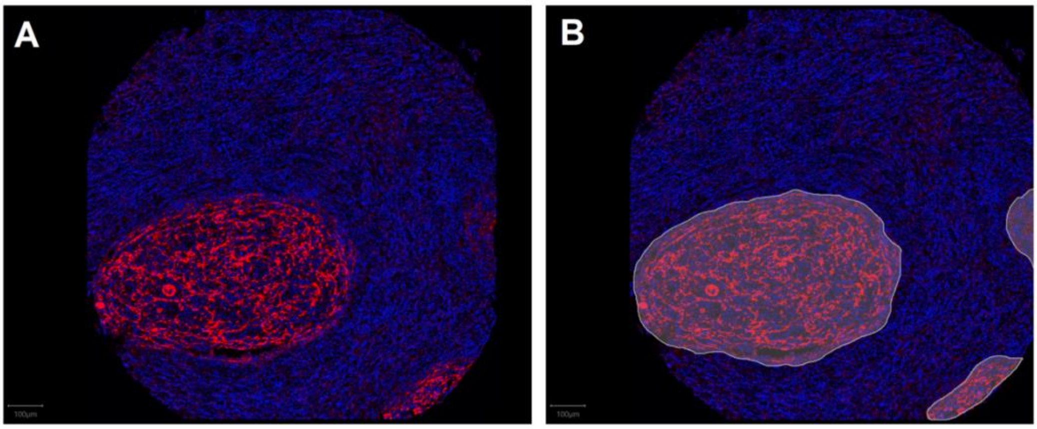

Fig. 6 Dendritic meshwork areas were annotated manually by drawing around the $CD21^+$ meshwork pattern regions. **A** CD21 (red) and DAPI (blue) view of a multi-plex TMA core image. **B** Manual annotation of dendritic meshwork areas, overlayed in grey.

6. Clinical characteristics of patients

Table 4 Baseline characteristics of the 127-patient cohort

Characteristic	Value	No.	%
Median Age, years	59		
Age, years	≤ 60	69	54%
	> 60	58	46%
Age Range, years	31-92		
Histologic Grading	1	37	29%
	1/2	12	9%
	2	45	35%
	2/3a	6	5%
	3a	20	16%
	Unspecified	7	6%
Serum LDH	> 549 IU/L	12	11%
	≤ 549 IU/L	96	89%
Ann Arbor Stage	I-II	46	36%
	III-IV	81	64%
No. of Nodal Sites	0-4	79	70%
	> 4	35	30%
Hb Level g/dL	≥ 12	91	82%
	< 12	20	18%
BM Involvement	Presence	45	38%
	Absence	73	61%
ENS, Excluding BM	Presence	37	30%
	Absence	88	70%
ECOG Performance Status	0-1	85	97%
	> 1	3	3%
FLIPI	0-1	45	44%
	2	33	33%
	3-5	23	23%
Initial Treatment	Watchful waiting (WW)	35	28%
	Radiotherapy	25	20%
	Rituximab regimens	67	52%
Rituximab Regimens	R-CVP	44	66%
	R-CHOP	9	13%
	R-Ibritumomab tiuxetan	10	15%
	Rituximab single agent	3	5%
	R-Bendamustine	1	1%

BM indicates bone marrow; ECOG, Eastern Cooperative Oncology Group (ECOG) Performance Status; ENS, Extra-Nodal Sites; Hb, haemoglobin; LDH, Lactic Acid Dehydrogenase; R, rituximab; R-CHOP, rituximab, cyclophosphamide, doxorubicin hydrochloride (hydroxydaunorubicin), vincristine sulphate and prednisone; and R-CVP, rituximab, cyclophosphamide, vincristine sulphate, and prednisone.

7. Prognostic value of POD24

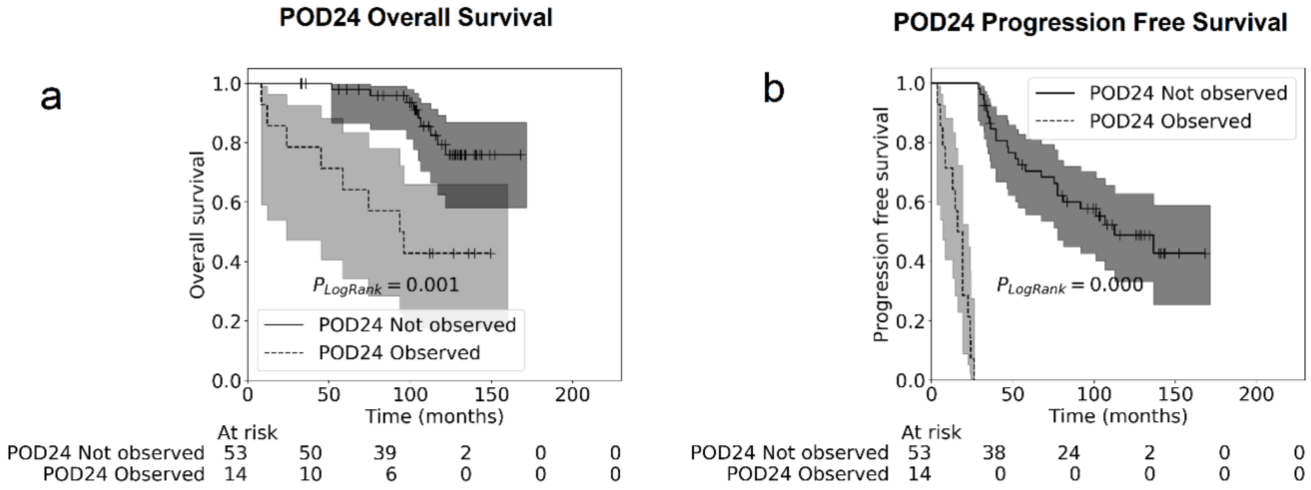

Fig. 7 Kaplan-Meier analysis with POD24 in the rituximab treated subgroup to test associations to OS (a) and PFS (b)

8. Prognostic value of patient clinical characteristics

Table 5 Survival and POD24 Analysis for Clinical Variables

Adverse Factor	Cox PH Univariable OS			Cox PH Univariable PFS			POD24	
	All Patients			Rituximab Patients				
	HR (95% CI)	P*	N	HR (95% CI)	P*	N	P_{POD24}†	N
Age > 60 years	2.80 (1.2, 6.53)	**0.017**	127	0.77 (0.41, 1.45)	0.412	67	0.088	67
Grade 3a, 3b	0.98 (0.36, 2.63)	0.961	120	0.61 (0.23, 1.57)	0.305	61	0.308	61
LDH > 549 IU/L	0.95 (0.22, 4.11)	0.942	108	0.82 (0.31, 2.14)	0.688	58	0.315	58
Stage III or IV	2.69 (0.99, 7.3)	0.052	127	2.18 (0.77, 6.15)	0.140	67	**0.041**	67
NS > 4	0.95 (0.37, 2.45)	0.912	114	1.29 (0.65, 2.55)	0.469	56	0.085	56
Hb < 12 g/dL	3.13 (1.23, 7.97)	**0.017**	111	2.66 (1.31, 5.43)	**0.007**	59	0.106	59
BM Presence	3.33 (1.39, 8.01)	**0.007**	118	1.78 (0.88, 3.6)	0.107	60	0.122	60
ECOG > 1	6.05 (0.73, 49.97)	0.095	88	6.83 (1.49, 31.39)	**0.014**	46	0.090	46
ENS Presence	3.81 (1.68, 8.63)	**0.001**	125	1.26 (0.67, 2.37)	0.474	65	0.445	65
FLIPI 0-5	1.57 (1.09, 2.26)	**0.014**	101	1.30 (0.95, 1.77)	0.102	51	0.214	51

BM indicates bone marrow; CI indicates confidence intervals; ECOG, Eastern Cooperative Oncology Group (ECOG) Performance Status; ENS, Extra-Nodal Sites; Hb, haemoglobin; HR, hazard ratio; LDH, Lactic Acid Dehydrogenase; NS, nodal sites; PH, proportional hazards; N, the number of patients.*P value testing significance of the log rank test. †P value testing significance of the Mann-Whitney U statistic testing differences between POD24 positive and negative subgroups. P values ≤ 0.05 are shown in bold.

FLIPI and extra-nodal site involvement predict OS

FLIPI was prognostic for overall survival (HR=1.57, 95% CI 1.09, 2.26) in the 101-patient cohort, but not PFS (HR=1.30, 95% CI 0.95, 1.77) in the 51 rituximab treated patients. When examining individual FLIPI components, age, stage and haemoglobin were associated with OS (Supplementary Table 5). Additionally, extra-nodal site (HR=3.81, 95% CI 1.68, 8.63) and bone marrow (HR=3.33, 95% CI 1.39, 8.01) involvement correlated to unfavourable OS.

Haemoglobin, ECOG status and stage predict early progression

Only low haemoglobin levels (HR=2.66, 95% CI 1.31, 5.43) and ECOG status (HR=6.83, 95% CI 1.49, 31.39) were associated with unfavourable PFS (Supplementary Table 5). Advanced stage at diagnosis was more commonly observed in patients who developed POD24 (p=0.041, Supplementary Table 5).

9. Distribution of tumour micro-environment features

Table 6 Median and interquartile range for tumour micro-environment features in the data set

Features		Feature Distribution (Median [Q25, Q75])		
		**Cohort (N=127)**	**Rituximab (N=67)**	**CoV**
**Cell Density, cells / mm²**	$CD4^+CD68^-$ T-helper cells	219.5 [110.9, 311.0]	170.6 [83.3, 275.9]	45.7%
	$CD4^+FOXP3^+$ T-regs	14.1 [5.8, 24.1]	11.5 [5.7, 23.8]	51.6%
	$CD8^+$ T-cells	72.8 [26.8, 125.5]	58.0 [22.8, 117.0]	37.4%
	$CD68^+$ cells	126.0 [77.6, 184.6]	121.2 [74.9, 171.8]	28.7%
	$CD4^+CD68^-PD\text{-}1^+$	26.6 [9.0, 58.3]	25.1 [6.7, 53.5]	61.3%
	$CD8^+PD\text{-}1^+$	10.3 [3.9, 23.0]	9.5 [3.9, 17.0]	58.3%
**Cell Ratio**	Immune infiltrate ratio†	0.4 [0.3, 0.7]	0.4 [0.2, 0.6]	32.4%
**% Positive Area**	$CD21^+$ dendritic meshwork area	0.3 [0.0, 0.4]	0.3 [0.1, 0.5]	73.5%
**Diversity, natural digits**	Phenotype entropy	1.9 [1.7, 2.1]	1.9 [1.8, 2.1]	8.3%
	Interaction entropy	4.0 [3.6, 4.4]	4.0 [3.7, 4.4]	7.7%

Q25 and Q75: 25th and 75th quantile, respectively. CoV: The average intra-patient coefficient of variation. †Immune infiltrate ratio is calculated as the total immune cells (positive for any marker) divided by the number of cells that expressed only DAPI.
